# The role of *cis*-elements in the evolution of crassulacean acid metabolism photosynthesis

**DOI:** 10.1038/s41438-019-0229-0

**Published:** 2020-01-01

**Authors:** Li-Yu Chen, Yinghui Xin, Ching Man Wai, Juan Liu, Ray Ming

**Affiliations:** 1 FAFU and UIUC Joint Center for Genomics and Biotechnology, Fujian Provincial Key Laboratory of Haixia Applied Plant Systems Biology, Key Laboratory of Genetics, Breeding and Multiple Utilization of Crops, Ministry of Education, Fujian Agriculture and Forestry University, 350002 Fuzhou, Fujian China; 2 Department of Plant Biology, University of Illinois at Urbana-Champaign, Urbana, IL 61801 USA

**Keywords:** Evolutionary biology, Plant evolution

## Abstract

Crassulacean acid metabolism (CAM) photosynthesis is an innovation of carbon concentrating mechanism that is characterized by nocturnal CO_2_ fixation. Recent progresses in genomics, transcriptomics, proteomics, and metabolomics of CAM species yielded new knowledge and abundant genomic resources. In this review, we will discuss the pattern of *cis*-elements in stomata movement-related genes and CAM CO_2_ fixation genes, and analyze the expression dynamic of CAM related genes in green leaf tissues. We propose that CAM photosynthesis evolved through the re-organization of existing enzymes and associated membrane transporters in central metabolism and stomatal movement-related genes, at least in part by selection of existing circadian clock *cis*-regulatory elements in their promoter regions. Better understanding of CAM evolution will help us to design crops that can thrive in arid or semi-arid regions, which are likely to expand due to global climate change.

## Introduction

**Fig. 1:**
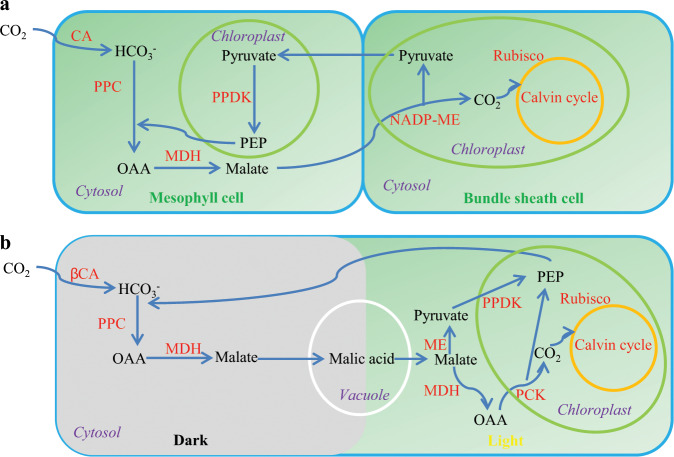
Photosynthetic reactions in C_4_ and CAM plants. **a** NADP-malic enzyme type of C_4_ pathway. **b** Carbon fixation in CAM plants.

Photosynthesis is the process that harvests solar energy to synthesize organic compounds that can ultimately be utilized to drive cellular processes by all forms of life. Photosynthesis is known from cyanobacteria to their descendants including algae and vascular plants^1^. There are three different photosynthetic pathways in terrestrial plants for fixation of carbon dioxide (CO_2_): C_3_, C_4_, and CAM. C_3_ photosynthesis is employed by most vascular plants. C_4_ plants represent about 3% of vascular plants^2^, while CAM plants represent about 6%^3^. Both C_4_ and CAM are add-ons to the C_3_ pathway. C_4_ and CAM metabolisms are similar in biochemistry but CO_2_ concentration steps are spatially separated in C_4_ rather than temporally as in CAM. C_4_ minimizes photorespiration by concentrating CO_2_ in bundle sheath cells, which relies in part on the unique cellular structure (Fig. 1a). Many C_4_ plants are agronomically important species, such as maize and sugarcane^4^. CAM plants have high water-use efficiency (WUE, expressed as mmol CO_2_ mol^−1^ H_2_O), which is a direct consequence of the fact that they open their stomata at night and keep them closed during the daytime^5^. WUE for carbon assimilation in CAM plants is much higher than in C_3_ or C_4_ plants. It will be 2–10 times higher than that of C_4_ plants and 2.6–20 times higher than that of C_3_ plants^6^. One of the major differences between C_4_ and CAM photosynthesis centers on the temporal regulation of CO_2_ absorption and fixation (Fig. 1).

CAM is found in over 400 genera across 36 families of vascular plants^7^ and has evolved multiple times independently from diverse ancestral C_3_ plants^3^. While we know ecological factors such as drought condition and CO_2_ concentration drive the evolution of CAM^8–10^, far less is known about genetics. Gene family duplication was previously proposed as the driver of CAM metabolism evolution through neofunctionalization of newly duplicated paralogous genes^3^, while others proposed that C_4_ and CAM photosynthesis may have arisen through the re-organization of metabolic processes already present in C_3_ plants^11–13^.

The past few years have seen the rapid progresses in genomics, transcriptomics, proteomics, and metabolomics for an increasing number of plant species including CAM species. The orchid *Phalaenopsis equestris* was the first CAM species for which the genome was assembled in 2015^14^. In the same year, the genome of fruit pineapple (*Ananas comosus* var. *comosus*) cultivar ‘F153’, which has been cultivated by Del Monte for 50 years, was sequenced, and the evolution of CAM photosynthesis was investigated^15^. In a following work, temporal and spatial transcriptomic profiles of CAM-performing mature leaves have also been studied in fruit pineapple^16^. Recently, we sequenced the *bracteatus* pineapple (*Ananas comosus* var. *bracteatus*) accession CB5 genome, and assembled to chromosomal level^17^. The genome of *Kalanchoë fedtschenkoi*, a eudicot CAM species was also available in 2017^18^. Furthermore, multi-dimensional omics data were available for CAM species, such as *Agave americana*^19,20^, ochids^21,22^, and *Talinum triangulare*^23^. These progresses for species that evolved CAM independently provide an excellent resource for comparative analyses, which will help us have a better understanding on the evolution of CAM photosynthesis.

## ***C****is*-elements of stomatal movement-related genes

Stomata were first described as ‘pore-like’ structures on the surface of leaves over three centuries ago^24^. Since the earliest examples of stomata were discovered in the leaf fossil record, plants have been evolving in terms of size and density of stomata to maintain the maximum leaf conductance as the atmosphere CO_2_ changed^25^. Stomata play an essential role in controlling of transpiration rate and water homeostasis in plants^26^. Stomatal movement can be stimulated by different environmental factors, such as light, abscisic acid (ABA), pathogens, CO_2_ and air humidity^27^. Among them, air humidity and ABA are directly related to water status in plants^28^. In CAM plants, the diel rhythms of stomatal conductance and transpiration are closely linked to the net CO_2_-uptake rhythm^5^.

CAM plants present a reverse stomatal conductance pattern by assimilating CO_2_ during the night when the temperature is low resulting in lower evapotranspiration rate compared to C_3_ and C_4_ plants^29,30^. This unique pattern of stomatal movement leads to the higher WUE in CAM plants^31^. The reverse stomatal rhythm has aroused curiosity and investigation for centuries^32^. Understanding the regulation of stomatal movement-related genes in CAM species may provide promising opportunities for engineering crops with higher WUE^32^.

We identified 118 stomatal movement-related genes in *A. comosus* var. *comosus*, 95 in *A. comosus* var. *bracteatus*, 121 in *P. equestris*, 140 in *Arabidopsis*, 123 in rice, and 121 in sorghum (Supplementary Table S1). Based on the GO annotation, the stomatal movement-related genes were divided into three categories, including genes involved in stomatal opening, stomatal closure, and regulation of stomatal movement. For genes involved in stomatal movement, the CIRCADIAN CLOCK ASSOCIATED 1 (CCA1)-binding site (CBS; AAAAATCT) and G-box binding site (CACGTG) showed more than 10% or higher frequency than the expected frequencies based on random chance in *A. comosus* var. *comosus* (Table 1). The G-box element was enriched in genes involved in all three categories in *A. comosus* var. *comosus* (Table 1). The evening element (EE; AAAATATC) and CBS were enriched in 123 stomatal movement-related genes in rice, whereas the morning element (MOE; CCACAC) was only enriched in stomatal opening category in rice (Table 1). When comparing with non-CAM species, Motif ERF73, ERF7, and ABR1 were enriched in CAM species (Supplementary Table S2). Based on these in silico findings, we propose the hypothesis that these different sets of *cis* elements regulate stomatal opening during the day and closure during the night for these three C_3_ and C_4_ species. Interestingly, stomatal movement-related genes in *A. comosus* var. *comosus* have higher frequencies of circadian clock *cis*-regulatory elements than *A. comosus* var. *bracteatus* (Table 1).

**Table 1:** Frequency of circadian clock-associated motifs (per kb) in 2 kb promoter regions of genes involved in stomatal movement^a^.

Circadian Motif	*A. comosus* var. *comosus*	*A. comosus* var. *bracteatus*	*P. equestris*	*Arabidopsis*	rice	sorghum
*Genes involved in stomatal movement*^b^						
MOE	0.212 (0.250)	0.170 (0.269)	0.132 (0.152)	0.164 (0.215)	0.333 (0.367)	0.281 (0.327)
EE	0.110 (0.105)	0.101 (0.096)	0.107 (0.168)	0.125 (0.124)	**0.106** (0.059)	0.074 (0.072)
CBS	**0.148** (0.105)	**0.160** (0.096)	0.087 (0.168)	0.118 (0.124)	**0.069** (0.059)	0.058 (0.072)
G-box	**0.288** (0.250)	0.250 (0.269)	0.120 (0.152)	0.232 (0.215)	0.317 (0.367)	0.248 (0.327)
*Regulation of stomatal movement*						
MOE	0.209	0.180	0.156	0.174	0.276	0.256
EE	0.115	0.090	0.065	0.107	**0.141**	**0.103**
CBS	**0.203**	**0.172**	0.097	0.112	**0.090**	0.064
G-box	**0.284**	0.270	0.130	**0.247**	0.359	0.231
*Stomatal closure*						
MOE	0.185	0.188	0.089	0.121	0.397	0.296
EE	0.093	0.094	**0.214**	0.136	**0.069**	0.037
CBS	0.074	**0.188**	0.071	**0.152**	0.034	0.074
G-box	**0.296**	0.000	0.089	0.152	0.259	0.352
*Stomatal opening*						
MOE	0.250	0.206	0.088	0.184	**0.471**	0.353
EE	0.111	**0.176**	0.118	**0.184**	0.000	0.000
CBS	0.028	0.088	0.059	0.079	0.029	0.000
G-box	**0.278**	0.235	0.118	**0.289**	0.206	0.147

^a^**Clock-associated motifs** with ≥10% frequency higher than expected frequency appearance in stomatal movement-related genes than expected genome wide frequency is highlighted in bold

^b^Species-specific expected frequencies are indicated in brackets. For example, in *A. comosus* var. *comosus*, since the genome GC content is 38%, G/C and A/T occurrence probabilities are 0.19 and 0.31. Both forward and reversed strands were included to calculate the expected frequency of the motif occurrence per kb (For MOE (CCACAC): 0.19 × 0.19 × 0.31 × 0.19 × 0.31 × 0.19 × 2 × 1,000 = 0.250)

In *A. americana*, the temporal re-programming of particular genes, including CO_2_ and ABA signaling and turgor pressure regulating genes are essential to regulate stomatal movement^19^. Comparative transcriptomic analyses between the C_3_ and CAM *Erycina* species also showed that genes involved in light and ABA signaling are altered^22^. The numbers of genes that contain *cis*-elements involved in several key stomatal movement pathways, such as light, ABA, and stress, are summarized (Table 2). ABA responsiveness-related motif (ABRE) appeared most frequently compared to other signaling pathways in the three species (*Arabidopsis*, *P. equestris*, and sorghum) with different photosynthetic pathways. From previous studies, exogenous ABA can induce stomatal closure and the expression and activity of CAM^33,34^. Moreover, stress-related motif (STRE) was the most frequent in the stomatal related genes of rice and pineapple (Table 2). The stress-induced stomatal movement signaling pathway is closely related to the water status of the plant^35^. Further genomic and molecular analysis of potential stomatal movement genes will enable us to have a comprehensive understanding of stomatal biology of CAM plants, and might provide candidate genes for engineering crop plants with higher sustainable production^32,36^.

**Table 2:** The number of genes and their percentages to the total genes of the genomes that contain *cis*-elements involved in partial key stomatal movement pathways annotated at promoter regions of orthologs in *A. comosus* var. *comosus*, *A. comosus* var. *bracteatus*, *P. equestris*, *Arabidopsis*, rice, and sorghum.

Pathways		*A. comosus* var. *comosus*	*A. comosus* var. *bracteatus*	*P. equestris*	*Arabidopsis*	rice	sorghum
Light responsiveness	Box 4^a^	107 (0.40%)	69 (0.23%)	30 (0.10%)	116 (0.24%)	89 (0.17%)	76 (0.22%)
	GT1-motif^b^	73 (0.27%)	42 (0.14%)	38 (0.13%)	101 (0.21%)	80 (0.15%)	61 (0.18%)
	GA-motif^c^	25 (0.09%)	11 (0.04%)	19 (0.06%)	29 (0.06%)	17 (0.03%)	33 (0.10%)
	G-Box^d^	40 (0.15%)	18 (0.06%)	36 (0.12%)	40 (0.08%)	50 (0.10%)	105 (0.31%)
	TCT-motif^e^	75 (0.28%)	46 (0.16%)	48 (0.16%)	112 (0.23%)	55 (0.10%)	56 (0.16)
ABA responsiveness	ABRE^f^	101 (0.37%)	65 (0.22%)	58 (0.20%)	123 (0.25%)	115 (0.22%)	112 (0.33%)
Stress	WUN-motif^g^	29 (0.11%)	27 (0.09%)	42 (0.14%)	62 (0.13%)	47 (0.09%)	43 (0.13%)
	STRE^h^	116 (0.43%)	89 (0 .30%)	35 (0.12%)	82 (0.17%)	116 (0.22%)	104 (0.30%)

^a^ Box 4-motif (ATTAAT)

^b^GT1-motif (GGTTAA)

^c^GA-motif (ATAGATAA)

^d^G-Box (CACGTG)

^e^TCT-motif (TCTTAC)

^f^ABRE-motif (ACGTG)

^g^WUN-motif (AAATTACT)

^h^STRE motif (AGGGG)

## Diurnal transcript abundance patterns of CAM pathway genes: pineapple as an example

The pineapple genome assembly also allowed the identification of full- and partial-length predicted amino-acid sequences of the key metabolic enzymes comprising the core carboxylation module of CAM responsible for nocturnal fixation of CO_2_^15,31,37^. Carbonic anhydrase (CA), catalyzing the conversion of CO_2_ into HCO_3_^–^, is responsible for the first step in CO_2_ assimilation both in C_4_ and CAM plants. All three CA subfamily (α, β, and γ) enzymes were identified in pineapple genome (Supplementary Table S3). Only *βCA* genes (*AccβCA2–1* and *AccβCA2–2*) implicated in CAM-specific roles due to their mRNA abundance in green leaf tissue^15^, indicating that βCA may acts as the enzyme in the initiation of CO_2_ fixation.

Three genes encoding the key enzyme PPC responsible for nocturnal CO_2_ fixation were identified in the genome assembly, all of which are predicted to be localized to the cytosol as expected^15^. Three PPC genes were identified in *comosus* pineapple genome (Supplementary Table S3, Supplementary Fig. S1). Among these three *PPC* genes, *AccPPC1* is the most abundant transcript (>3000 FPKM, fragments per kilobase of exon per million fragments mapped) and displayed highest abundance at 6 pm (>5500 FPKM) in CAM-performing leaf tissues. In *T. triangulare*, a facultative CAM species, *PPC* was upregulated 25-fold (to 15,510 rpm, reads per million) at midnight on day 9 and 12 of water limitation when indicative of CAM was observed^23^. Comparative transcriptomic analyses between the C_3_ and CAM *Erycina* species also demonstrated that *PPC* gene in CAM *Erycina* displayed higher abundance than in C3 *Erycina*^22^. These results suggest that high levels of *PPC* transcripts are important for CAM.

PPC undergoes reversible N-terminal phosphorylation by a circadian clock-controlled PPC kinase (PPCK), which reduces the sensitivity of the enzyme to allosteric inhibition by L-malate and increases its affinity for its substrate phospho*enol*pyruvate (PEP)^38,39^. In *A. americana*, which is an obligate CAM plant, *PPCK1* gene displayed diel transcripts abundance pattern, suggesting its important role in temporal re-programming of CAM^20^. In *K. fedtschenkoi*, PPCK1 is also essential for nocturnal CO_2_ fixation; moreover, knock-down of oscillations in the transcript abundance of *PPCK1* will lead to the altered accumulation and periodicity of core circadian clock-related transcripts^40^. In pineapple, *AccPPCK2* was found to exhibit greater mRNA abundance than *AccPPCK1*, and *AccPPCK2* also displayed diel mRNA abundance with high levels at night, suggesting that it functions in CAM^15^.

In the final metabolic step of phase I, the OAA formed as a result of PEP carboxylation is reduced to malate by NAD(P)-dependent malate dehydrogenase (MDH). Fourteen genes in pineapple encode MDH: three genes (*AccMDH4*, *AccMDH5*, and *AccMDH8*) are predicted to be cytosolic-localized and strongly expressed in leaves, suggesting their potential to perform functional roles in CAM; four genes (*AccMDH10*, *AccMDH11*, *AccMDH12*, and *AccMDH13*) are tandemly duplicated and lowly expressed except *AccMDH13*^15^.

In *Arabidopsis*, the malate is transported into the vacuole by an inward-rectifying anion-selective ion channel belonging to the aluminium-activated malate transporter (ALMT) family^41^. In *K. fedtschenkoi*, a putative *ALMT6* gene (Kaladp0062s0038) displays diel mRNA abundance in leaves^18^. There are eight candidate *ALMT* family genes in pineapple, including three *ALMT9* genes (*AccALMT9-1–3*) and five *ALMT1* genes (*AccALMT1-1*–*5*). Only two *ALMT9* genes (*AccALMT9-1* and *AccALMT9-3*) showed high abundant transcript levels in photosynthetic leaf tissues. *ALMT1* only has higher steady-state transcript levels at the midday on day 9 of water limitation in *T. triangulare*^23^. The malate then undergoes protonation, with protons supplied by the tonoplast H^+^-ATPase and H^+^-PPiase, and is stored as malic acid. In the daytime, malic acid is effluxed out of the vacuole possibly through a putative tonoplast dicarboxylate transporter (tDT)^42^. There are five *DT* genes (*AccDT1*–*5*) in the pineapple genome, and *AccDT2* and *AccDT3* display specifically high abundant transcripts in daytime in photosynthetic leaf tissues, indicating that they may play a role in malic acid efflux in CAM. Decarboxylation of the malate during phase III of the CAM cycle occurs in pineapple primarily via PEP carboxykinase (PCK)^30,43^, which, following oxidation of malate to OAA by NAD(P)-dependent MDH, decarboxylates OAA to PEP. A single *PCK* gene (*AccPCK1*) is present in the pineapple genome and it is predicted to encode a cytosolic enzyme^15^. It is an ortholog of *AtPCK1* (AT4G37870.1), one of two *PCK* genes in *Arabidopsis*, which is expressed in guard cells and is implicated in stomatal closure^44^. Despite the fact that extractable PCK activity from pineapple leaves is over 15 times higher than that of the malic enzymes (MEs)^45^, and it remains possible that malate may also be decarboxylated, in part, by ME in pineapple^46^. The *comosus* pineapple genome contains five *ME* genes encoding both NAD- and NADP-ME (Supplementary Table S3): two *NADP-ME* genes (*AccNADP-ME1* and *AccNADP-ME3*) exhibit higher mRNA levels during the daytime in photosynthetic leaf tissues and one additional *NADP-ME* gene (*AccNADP-ME2*) shows none mRNA transcript in leaves; two *NAD-ME* genes (*AccNAD-ME1* and *AccNAD-ME2*) encoding isoforms predicted to be localized to the mitochondria exhibit moderate abundant mRNA expression and *AccNAD-ME2* also displayed higher mRNA level during the daytime^15^.

The abundant transcript level for *ME* genes in pineapple suggests that malate decarboxylation also results in the formation of pyruvate, which must then be phosphorylated to PEP by pyruvate phosphate dikinase (PPDK). Consistent with this supposition, a single candidate *PPDK1* gene (*AccPPDK1*) was identified in the pineapple genome^15^, providing the metabolic flexibility to allow gluconeogenesis via both the PCK and ME/PPDK routes^47^. *AccPPDK1* displayed higher transcript abundance during the daytime. The *AtPPDK1* gene encodes an enzyme predicted to be localized to the cytosol, but this enzyme might be localized to either the chloroplast or the cytosol depending upon the production of alternative transcripts arising from two different promoters^48^. More detailed examination of this locus in pineapple is needed to verify this possibility. Overall, the enzymes making up the carboxylation and decarboxylation pathways in the CAM cycle in pineapple are encoded by gene families that are generally smaller than those encoded by the *A. thaliana* genome, because pineapple has one fewer whole-genome duplications than that have been reported for *Arabidopsis* and the grass family^49^.

## Circadian clock-associated *cis*-elements in CAM genes

In most living organisms, internally synchronized circadian clocks make it possible for them to coordinate behavior and physiology corresponding with the 24 h light-dark cycle. CCA1 and LATE ELONGATED HYPOCOTYL (LHY), two single-MYB domain transcription factors, are central to the circadian oscillator of angiosperms^50,51^. *CCA1* and *LHY* are morning expressed genes. They act to suppress the expression of the DNA sequence they bind to. CCA1 and LHY are partially redundant, and they can directly bind to the *TIMING OF CAB EXPRESSION 1* (*TOC1*) also known as *PRR1* (*PSEUDO-RESPONSE REGULATOR 1*) promoter to negatively regulate its expression^52^.

Circadian control of CAM has been implicated as a core component in diel re-programming of metabolism in CAM plants^20,53^. A comprehensive spatial and temporal survey of gene co-expression clusters in pineapple leaf tissues reveals CAM pathway genes are enriched with clock-associated *cis*-elements, suggesting circadian regulation of CAM^15,16^. At dawn, CCA1 and LHY repress evening-phased genes by binding to CBS and EE^49^. In addition to CBS and EE, the G-box is also enriched in the CCA1 binding regions^54,55^. TOC1 can bind to MOE as a negative regulator^56^. In pineapple, all of the three *βCA* genes contain CBS in their promoter regions (Table 3), suggesting they may have function in *βCA* genes’ nighttime and early-morning transcripts abundance pattern in photosynthetic leaf tissues. All three copies of *PPC* genes also contain CBS in their promoter regions, along with MOE or G-box (Table 3). Interestingly, CAM pathway genes in *A. comosus* var. *comosus*, contain more circadian clock *cis*-regulatory elements than *A. comosus* var. *bracteatus* (Table 3). Besides the core CAM genes, more than 40% of transcription factors and transcription co-regulators displayed diel rhythmic expression in pineapple, suggesting it is a global adaptation^57^. In a recent work by Heyduk and colleagues (2018), they demonstrated that some canonical CAM genes were unaltered by comparative transcriptomic analyses between the C_3_ and CAM *Erycina* species. However, 149 gene families, including genes involved in light and ABA signaling, had significant differences in network connectivity, indicating that transcriptional cascades changes are critical for the transition from C_3_ to CAM in *Erycina*^22^.

**Table 3:** *Cis*-elements annotated at promoter regions of selected CAM photosynthetic genes in pineapple.

CAM enzyme	*A. comosus* var. *comosus*		*A. comosus* var. *bracteatus*	
	Gene ID	TF binding motif	Gene ID	TF binding motif
βCA	Aco002732	CBS (2)	CB5.v30091940	CBS (2), G-Box (3)
	Aco005402	CBS (1)	CB5.v30297520	CBS (2)
	Aco006181	CBS (2)	CB5.v30069370	–
PPC	Aco010025	EE (1), MOE (2), CBS (2)	CB5.v30072230	–
	Aco018093	MOE (2), CBS (1)	CB5.v30160110	EE (1), MOE (1)
	Aco016429	G-box (1), CBS (1)	CB5.v30185780	–
			CB5.v30098990	G-box (1)
PPCK	Aco010095	G-box (1)	CB5.v30291670	–
	Aco013938	G-box (1)	CB5.v30308180	G-box (3)
MDH	Aco006122	CBS (1)	CB5.v30030450	–
	Aco007734	CBS (2)	CB5.v30063990	–
	Aco013935	MOE (2)	CB5.v30119070	–
	Aco002885	MOE (2), G-box (1)	CB5.v30217860	–
	Aco004349	MOE (2), G-box (1)	CB5.v30283140	CBS (2), G-box (2)
	Aco014690	G-box (2)	CB5.v30016950	CBS (1)
	Aco017525	CBS (3)	CB5.v30081100	MOE (1)
	Aco017526	–	CB5.v30308160	MOE (2)
	Aco017527	CBS (1)	CB5.v30167990	–
	Aco017528	CBS (2)	CB5.v30057750	EE (1), CBS (1)
	Aco019631	CBS (1)	CB5.v30057770	–
	Aco010232	MOE (1), G-box (1)	CB5.v30175150	CBS (1)
	Aco004996	MOE (3)	CB5.v30175160	CBS (2)
	Aco008626	–	CB5.v30057740	CBS (1)
			CB5.v30057760	G-box (1)
NAD_ME	Aco016569	–	CB5.v30106390	–
	Aco007622	CBS (1), G-box (1)	CB5.v30132860	CBS (1), G-box (2)
NADP_ME	Aco009967	G-box (4), MOE (1)	CB5.v30038850	G-box (2)
	Aco005631	CBS (1)	CB5.v30285200	CBS (1)
	Aco005989	G-box (2)	CB5.v30300470	G-box (3), ME (1)
PCK	Aco017762	MOE (2), G-box (1), CBS (1)	CB5.v30124740	MOE (1), G-box (1), CBS (1)
PPDK	Aco024818	EE (2), G-box (1), CBS (1)	CB5.v30137360	EE (1), G-box (1), CBS (1)

## Evolution of CAM photosynthesis

C_4_ and CAM photosynthesis are innovations that evolved in response to decreasing atmospheric levels of CO_2_ and water-limiting environments^2,9^. CAM has a higher incidence^3^, and mutation of CAM genes in CAM species is not lethal^40,58^. Both C_4_ and CAM have evolved independently multiple times, even within individual families, or even genera during angiosperm evolution^59–61^. For example, in the Neotropical family Bromeliaceae, to which pineapple belongs, CAM photosynthesis evolved independently at least four, and probably five times^59^.

Recruitment of pre-existing mechanisms underlying C_3_ photosynthesis is adopted in *Gynandropsis gynandra* (referred to previously as *Cleome gynandra*), a C_4_ plant which is relatively closely related to *Arabidopsis*^62^. Furthermore, gene duplication also plays a profound role in the evolution of C_4_. For example, *βCA* genes are tandemly duplicated in sorghum^63^. After duplication, some C_4_ genes, such as C_4_ PPC genes, NADP-MDH genes, and PPDK genes, underwent adaptive evolution^63^.

Comparative analyses demonstrated signatures of convergence in protein sequence and re-scheduling of diel transcript abundance of genes involved in nocturnal CO_2_ fixation, stomatal movement, heat tolerance, the circadian clock, and carbohydrate metabolism^14,18,21^. Firstly, convergent evolution has been detected in terms of diel cycles of gene transcript abundance^18^. PPCK is a key regulator of PPC, which can activate PPC by phosphorylating it. Both *AccPPCK2* and *KfPPCK2* showed diel expression patterns^18^. Secondly, a convergent amino-acid change in PPC2 was discovered to be shared by *K. fedtschenkoi* and *P. equestris* and the *PPC2* gene in *K. fedtschenkoi* is a much lower abundance transcript relative to the CAM-associated *PPC1* gene, so the function of PPC2 has yet to be linked to CAM directly in either *K. fedtschenkoi* or *P. equestris*^18^.

These findings are consistent with the hypothesis that the CAM photosynthesis evolved as a result of a re-organization of pre-existing metabolic pathways^11,15^. These different features were later coordinated to form the functional CAM photosynthesis.

## Concluding remarks

Genomic studies have led to a renaissance in CAM research. Recent genomic and transcriptomic information from CAM species has improved our understanding of the evolution of CAM photosynthesis^14–21^. The identified candidate genes provide initial targets for detailed functional studies of how the CAM genes have evolved through regulation of gene expression to gain the observed spatial and temporal expression patterns, and loss of repressors is certainly involved. It may be possible for us to apply genome editing to verify functions of candidate CAM genes. CRISPR/Cas9 technology will be a powerful tool to get higher order mutants of tandemly duplicated genes in the same chromosome, which is impossible to generate by traditional mutagenesis methods.

Water loss from stomata for C_3_ plants can be very substantial under hot and dry condition. Adjusting the temporal pattern of stomatal movement genes may be a key evolutionary step for switching stomatal opening from the light period to dark^32^. Enrichment of different sets of circadian clock regulatory *cis* elements may have played a role in this dramatic shift in gene regulation in pineapple and *P. equestris*. CAM photosynthesis and its associated high WUE are key evolutionary innovations that adapted to arid environments and/or low CO_2_ environment and this valuable trait is a direct consequence of stomatal closure throughout hottest and driest part for the 24 h cycle, and leaf succulence.

## Supplementary information

Supplementary Note

Supplementary Figure 1

Supplementary Table 1-3
