# Supplementary material for: The role of cis-elements in the evolution of crassulacean acid metabolism photosynthesis
Source: Hortic Res. 2020 Jan 1;7:5. doi: 10.1038/s41438-019-0229-0 (PMC6938490; doi:10.1038/s41438-019-0229-0)
Supplement: Supplementary file 1 — Supplementary Note [file 41438_2019_229_MOESM1_ESM.docx]

**The role of *cis*-elements in the evolution of crassulacean acid metabolism photosynthesis**

**Supplementary Note**

**Materials and Methods**

**Identification of stomatal movement-related genes**

The latest genomes of *Arabidopsis thaliana*^1^, *Ananas comosus* var. *comosus*^2^, *Ananas comosus* var. *bracteatus*^3^, *Phalaenopsis equestris*^4^, *Oryza sativa*^5^, and *Sorghum bicolor*^6^ were used for analysis. 140 genes with stomatal movement-related Gene Ontology annotation (obtained from The Arabidopsis Information Resource (TAIR) <https://www.arabidopsis.org/index.jsp>) in *A.* *thaliana* were used as the query to identify orthologs in other species. Stomatal movement-related orthologs in *A. comosus* var. *comosus*, *A. comosus* var. *bracteatus*, *P. equestris*, rice, and sorghum were identified by using BLASTP (<https://blast.ncbi.nlm.nih.gov/Blast.cgi?PAGE=Proteins>) with a cutoff E-value of 1e-5 when comparing to stomatal movement-related genes in *A. thaliana*.

**Analysis of *cis*-elements in promoter region**

Based on GO annotation, the stomatal movement-related genes were divided into three categories, including genes involved in stomatal opening, stomatal closure, and regulation of stomatal movement. The circadian clock *cis*-regulatory elements in the 2 kb promotors of these genes were identified and analyzed. We examined the expected and actual frequencies of those clock-associated motifs in the 2 kb promoter regions in stomatal movement-related genes across the six species. We explored enriched cis-regulatory elements in the 2kb upstream promoter regions of stomatal movement-related genes in different photosynthetic types by using program CentriMo under MEME^7^. *Arabidopsis* DAP motif database^8^ was used to test the enrichment. The top 20 ranked motifs from the output were chosen for this study.

**References**

1 Lamesch, P. *et al.* The Arabidopsis Information Resource (TAIR): improved gene annotation and new tools. *Nucleic Acids Res.* **40**, D1202-1210, (2012).

2 Ming, R. *et al.* The pineapple genome and the evolution of CAM photosynthesis. *Nat. Genet.* **47**, 1435-1442, (2015).

3 Chen, L. Y. *et al.* The bracteatus pineapple genome and domestication of clonally propagated crops. *Nat. Genet.* **51**, 1549-1558, (2019).

4 Cai, J. *et al.* The genome sequence of the orchid Phalaenopsis equestris. *Nat. Genet.* **47**, 65-72, (2015).

5 Ouyang, S. *et al.* The TIGR Rice Genome Annotation Resource: Improvements and new features. *Nucleic Acids Res.* **35**, D883-D887, (2007).

6 McCormick, R. F. *et al.* The Sorghum bicolor reference genome: improved assembly, gene annotations, a transcriptome atlas, and signatures of genome organization. *Plant J.* **93**, 338-354, (2018).

7 Bailey, T. L. & Machanick, P. Inferring direct DNA binding from ChIP-seq. *Nucleic Acids Res.* **40**, (2012).

8 O'Malley, R. C. *et al.* Cistrome and epicistrome features shape the regulatory DNA landscape. *Cell* **166**, 1598-1598, (2016).
