# Supplementary figures and images for: The role of cis-elements in the evolution of crassulacean acid metabolism photosynthesis
Source: Hortic Res. 2020 Jan 1;7:5. doi: 10.1038/s41438-019-0229-0 (PMC6938490; doi:10.1038/s41438-019-0229-0)

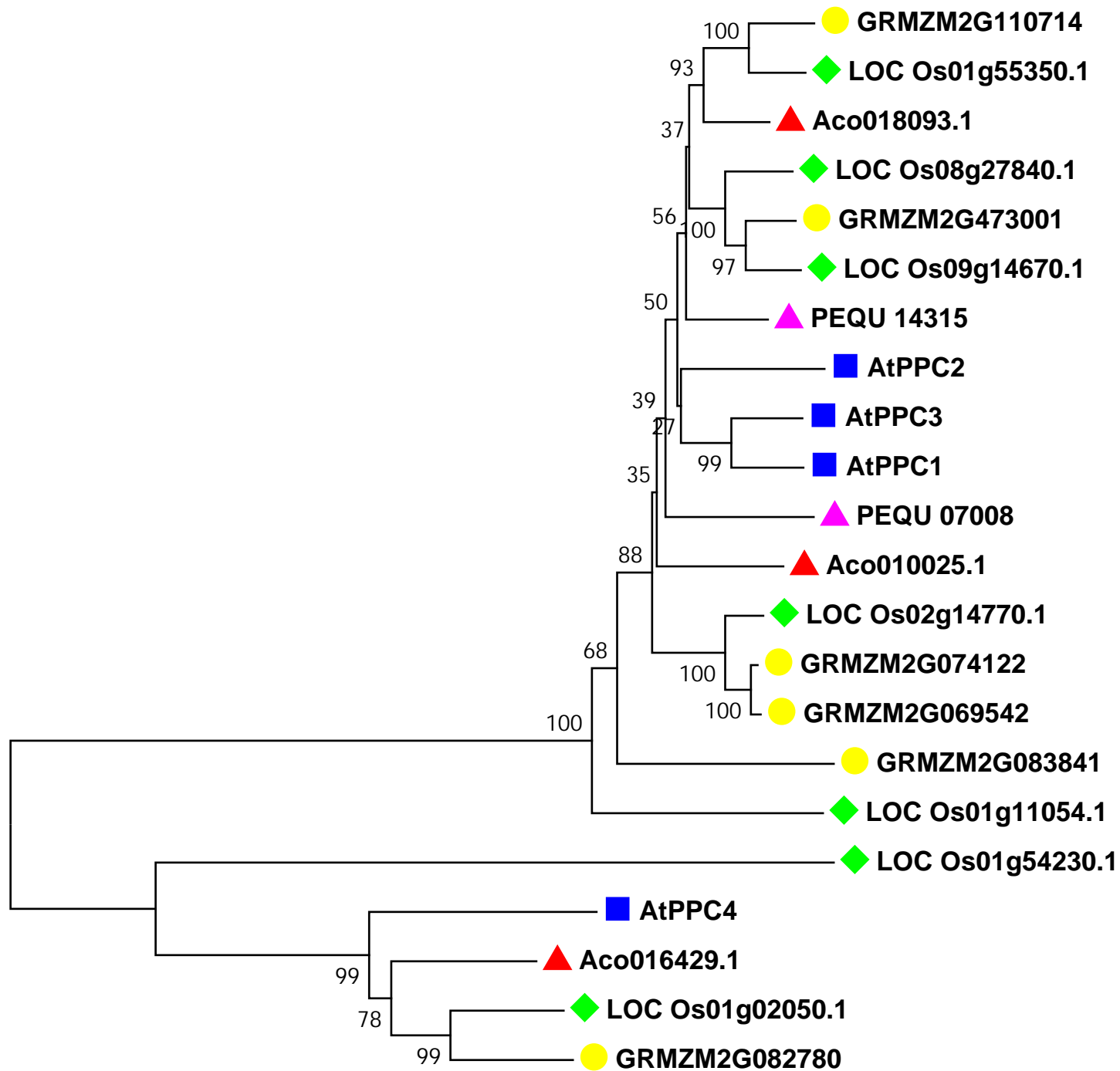

0.1

Supplement: Supplementary file 2 — Supplementary Figure 1 [file 41438_2019_229_MOESM2_ESM.pdf]
