# Supplementary material for: The role of cis-elements in the evolution of crassulacean acid metabolism photosynthesis
Source: Hortic Res. 2020 Jan 1;7:5. doi: 10.1038/s41438-019-0229-0 (PMC6938490; doi:10.1038/s41438-019-0229-0)
Supplement: Supplementary file 3 — Supplementary Table 1-3 [file 41438_2019_229_MOESM3_ESM.docx]

**Supplementary Table S1. Circadian clock *cis*-elements annotated at promoter regions of orthologs in *A. comosus* var. *comosus*, *A. comosus* var. *bracteatus*, *P. equestris*, *Arabidopsis*, rice, and sorghum.**

| Process | **Gene** | ***A. comosus* var. *comosus*** | | ***A. comosus* var. *bracteatus*** | | ***P. equestris*** | | ***Arabidopsis*** | | **rice** | | **sorghum** | |
| --- | --- | --- | --- | --- | --- | --- | --- | --- | --- | --- | --- | --- | --- |
|  |  | **Gene ID** | **TF binding motif** | **Gene ID** | **TF binding motif** | **Gene ID** | **TF binding motif** | **Gene ID** | **TF binding motif** | **Gene ID** | **TF binding motif** | **Gene ID** | **TF binding motif** |
| Stomatal opening | ABCC5 | Aco010163 | MOE | CB5.v30290980 | MOE | PEQU_13968 | MOE | AT1G04120 | G-box | LOC_Os03g04920 | MOE | Sobic.001G508200 |  |
|  | MAPKKK18 | Aco019577 | G-box |  |  | PEQU_06047 | MOE,G-box | AT1G05100 | EE,G-box | LOC_Os02g21700 |  | Sobic.003G268700 | MOE |
|  | OZS1 | Aco015541 | MOE | CB5.v30142270 | CBS | PEQU_13865 | MOE | AT1G12480 | EE,CBS | LOC_Os04g48530 | MOE,G-box | Sobic.006G186500 | G-box |
|  | UBC34 | Aco012146 | EE |  |  | PEQU_11913 | MOE | AT1G17280 | G-box | LOC_Os06g09330 | MOE,G-box | Sobic.010G071200 |  |
|  | CPK6 | Aco011911 | G-box | CB5.v30095690 | G-box | PEQU_00733 | MOE | AT2G17290 | G-box | LOC_Os04g49510 | MOE | Sobic.006G192500 |  |
|  | HA1 | Aco008192 |  | CB5.v30175810 |  | PEQU_17563 | MOE,G-box | AT2G18960 |  | LOC_Os04g56160 | MOE,G-box | Sobic.006G247100 |  |
|  | PSB29 | Aco009132 | G-box | CB5.v30189260 | G-box | PEQU_28231 | MOE,CBS | AT2G20890 | MOE,G-box | LOC_Os07g37250 | G-box | Sobic.002G338100 | G-box |
|  | PK1B | Aco014181 |  | CB5.v30273930 |  | PEQU_12478 | EE,MOE,CBS | AT2G28930 |  | LOC_Os03g60710 | MOE, | Sobic.001G033400 |  |
|  | UBC32 | Aco006362 |  | CB5.v30286840 |  | PEQU_41132 | MOE,G-box | AT3G17000 |  | LOC_Os03g19500 | G-box | Sobic.001G395400 | MOE |
|  | BLUS1 | Aco024455 | MOE,CBS | CB5.v30194150 | MOE,CBS | PEQU_03042 | EE,MOE | AT4G14480 | G-box | LOC_Os03g47470 | MOE | Sobic.008G011200 |  |
|  | RHC1 | Aco015732 | MOE,G-box | CB5.v30141140 |  | PEQU_15643 | MOE | AT4G22790 | MOE | LOC_Os03g64150 | G-box | Sobic.001G003700 |  |
|  | OST1 | Aco011646 |  | CB5.v30088390 |  | PEQU_27381 | MOE | AT4G33950 |  | LOC_Os03g41460 |  | Sobic.001G168400 | MOE |
|  | RZPF34 | Aco014646 | EE,G-box | CB5.v30053930 |  | PEQU_13602 | MOE,G-box | AT5G22920 | MOE,CBS | LOC_Os03g05270 |  | Sobic.001G505300 | MOE |
|  | CYCH;1 | Aco006781 | G-box | CB5.v30072630 |  | PEQU_37940 | MOE | AT5G27620 |  | LOC_Os03g52750 | MOE | Sobic.001G098100 | MOE |
|  | ERA1 | Aco006174 | MOE | CB5.v30069300 | MOE | PEQU_32125 | MOE | AT5G40280 | EE,CBS | LOC_Os01g53600 | CBS | Sobic.003G289400 | MOE,G-box |
|  | TOD1 | Aco010656 | EE | CB5.v30072110 |  | PEQU_19338 | MOE | AT5G46220 | MOE | LOC_Os01g11220 | MOE,G-box | Sobic.003G022400 |  |
|  | UBC33 |  |  | CB5.v30094440 |  |  |  | AT5G50430 | EE |  |  |  |  |
|  | HCF106 | Aco010606 | MOE,G-box | CB5.v30007300 | G-box | PEQU_27411 | EE,MOE | AT5G52440 | MOE,G-box | LOC_Os11g37130 |  | Sobic.006G055800 | MOE,G-box |
|  | ABI2 | Aco009886 | G-box | CB5.v30125730 | G-box |  |  | AT5G57050 | EE |  |  |  |  |
| Stomatal closure | EXO70B2 | Aco007086 | EE |  |  | PEQU_15985 | MOE | AT1G07000 |  | LOC_Os05g39610 | MOE | Sobic.003G341700 |  |
|  | BON3 | Aco002397 |  | CB5.v30158650 | MOE | PEQU_30092 | EE,MOE | AT1G08860 | G-box | LOC_Os05g30970 | G-box | Sobic.009G118300 | G-box |
|  | OZS1 | Aco015541 | MOE |  |  | PEQU_13865 | MOE | AT1G12480 | EE,CBS | LOC_Os04g48530 | MOE,G-box | Sobic.006G186500 | G-box |
|  | ALMT4 | Aco003023 | CBS,G-box | CB5.v30150580 | MOE,CBS,G-box | PEQU_32467 | MOE | AT1G25480 | EE,MOE | LOC_Os02g49790 |  | Sobic.010G112300 | G-box |
|  | [NOGC1](http://amigo.geneontology.org/amigo/gene_product/TAIR:locus:2203911) | Aco012064 | G-box | CB5.v30084010 | CBS,G-box | PEQU_17893 | MOE | AT1G62580 | EE | LOC_Os10g40570 | MOE,G-box | Sobic.001G303200 |  |
|  | FAB1C | Aco001051 | EE,CBS,G-box |  |  | PEQU_14451 | MOE | AT1G71010 |  | LOC_Os08g33200 |  | Sobic.007G133000 |  |
|  | LHCB2.2 | Aco026888 | G-box | CB5.v30051310 | G-box | PEQU_05059 | MOE,G-box | AT2G05070 |  | LOC_Os03g39610 | MOE,G-box | Sobic.001G177000 | MOE,G-box |
|  | KT1 | Aco005466 |  |  |  | PEQU_26896 | MOE | AT2G26650 |  | LOC_Os01g45990 | G-box | Sobic.003G237900 | MOE,G-box |
|  | TEJ | Aco018002 | CBS |  |  | PEQU_03354 | EE,MOE | AT2G31870 |  | LOC_Os03g62680 | MOE,G-box | Sobic.001G016000 | MOE,CBS,G-box |
|  | EULS3 | Aco018660 | G-box | CB5.v30192810 | G-box | PEQU_00754 | EE,MOE | AT2G39050 |  | LOC_Os07g48500 |  | Sobic.002G421300 | MOE |
|  | WRKY54 | Aco028684 | MOE |  |  | PEQU_18628 | MOE,CBS, | AT2G40750 | EE,CBS | LOC_Os05g25770 | MOE | Sobic.008G060300 |  |
|  | NHX2 | Aco001941 | MOE |  |  | PEQU_12382 | MOE,G-box | AT3G05030 |  | LOC_Os07g47100 | MOE,G-box | Sobic.002G408100 | EE,G-box |
|  | FAB1B | Aco021078 | MOE,G-box | CB5.v30250030 | MOE | PEQU_22419 | MOE | AT3G14270 | EE | LOC_Os03g28140 | MOE | Sobic.002G196600 | G-box |
|  | TCTP1 | Aco000662 | CBS | CB5.v30018830 | CBS | PEQU_15296 | MOE,CBS | AT3G16640 |  | LOC_Os11g43900 | CBS,G-box | Sobic.004G006300 | G-box |
|  | ZIFL2 | Aco019261 | EE |  |  | PEQU_23228 | EE,MOE | AT3G43790 |  | LOC_Os12g03899 | MOE | Sobic.005G024800 | MOE,G-box |
|  | MAPKKK20 |  |  |  |  | PEQU_27099 |  | AT3G50310 | EE |  |  |  |  |
|  | WRKY70 |  |  |  |  | PEQU_17654 | MOE | AT3G56400 | EE,MOE |  |  |  |  |
|  | SPP1 | Aco020413 |  | CB5.v30070240 |  | PEQU_25742 | MOE,CBS, | AT3G58490 |  | LOC_Os03g59070 | G-box | Sobic.001G049300 | MOE |
|  | LecRK-V.5 | Aco008603 |  |  |  | PEQU_33417 | EE,MOE,G-box | AT3G59700 | CBS | LOC_Os07g03830 | EE | Sobic.002G107900 | MOE |
|  | PCAP1 | Aco010808 | MOE,G-box | CB5.v30082310 | G-box | PEQU_20485 | MOE | AT4G20260 | EE,MOE,CBS,G-box | LOC_Os02g18410 | G-box | Sobic.004G128600 | MOE |
|  | CES1 | Aco007458 |  | CB5.v30012250 | CBS | PEQU_18209 | MOE | AT4G22330 | CBS | LOC_Os03g49180 | MOE | Sobic.001G127000 | EE,MOE |
|  | HXK1 | Aco005979 | G-box | CB5.v30285250 |  | PEQU_09766 | EE,MOE | AT4G29130 | MOE | LOC_Os05g45590 | MOE | Sobic.009G203500 | MOE,G-box |
|  | PLDDELTA | Aco013079 | MOE | CB5.v30212720 |  | PEQU_15635 | MOE | AT4G35790 | MOE | LOC_Os09g37100 |  | Sobic.002G282500 | MOE,CBS,G-box |
|  | HSC70-1 | Aco001457 |  | CB5.v30157690 |  | PEQU_18080 | MOE | AT5G02500 | MOE,G-box | LOC_Os11g47760 | EE | Sobic.008G136000 | G-box |
|  | HK5 | Aco003757 | EE,MOE |  |  | PEQU_15548 | MOE | AT5G10720 | CBS,G-box | LOC_Os06g44410 | EE,MOE,CBS | Sobic.010G213600 |  |
|  | ZIF1 |  |  |  |  |  |  | AT5G13740 | MOE,CBS,G-box | LOC_Os11g04020 | MOE |  |  |
|  | ZIFL1 |  |  | CB5.v30246860 |  |  |  | AT5G13750 | CBS | LOC_Os11g04104 | MOE,G-box |  |  |
|  | CaS | Aco003034 | MOE,G-box | CB5.v30150670 |  | PEQU_27284 | MOE | AT5G23060 |  | LOC_Os02g49680 | MOE,G-box | Sobic.004G251500 | CBS |
|  | NHX1 |  |  | CB5.v30166770 |  |  |  | AT5G27150 |  |  |  |  |  |
|  | ERF96 | Aco010430 | G-box |  |  | PEQU_17473 | MOE,G-box | AT5G43410 | G-box | LOC_Os09g39810 | EE | Sobic.002G139300 | G-box |
|  | HSP81-2 | Aco000816 |  | CB5.v30280580 |  | PEQU_15042 | MOE,CBS | AT5G56030 |  | LOC_Os09g30412 | G-box | Sobic.007G216300 | CBS |
|  | EXO70B1 |  |  |  |  |  |  | AT5G58430 |  | LOC_Os01g61180 | MOE |  |  |
|  | SCAP1/MBM17.11 | Aco019361 | EE |  |  |  |  | AT5G64010 | EE |  |  | Sobic.009G114900 | MOE |
| Regulation of stomata movement | CRY2 | Aco008958 | MOE | CB5.v30077680 | MOE,CBS | PEQU_22307 | MOE | AT1G04400 |  | LOC_Os02g41550 |  | Sobic.010G138000 | MOE |
|  | AKS2 | Aco011419 | MOE |  |  | PEQU_26890 | MOE | AT1G05805 |  | LOC_Os01g67480 | EE,MOE,G-box | Sobic.003G391100 | MOE,CBS |
|  | MYB60 | Aco017875 | EE | CB5.v30030350 | MOE | PEQU_10683 | MOE | AT1G08810 | MOE | LOC_Os12g03150 | EE,MOE | Sobic.008G020300 |  |
|  | MYB61 | Aco006386 |  |  |  | PEQU_20514 | EE,MOE,G-box | AT1G09540 |  | LOC_Os05g04820 | MOE,CBS | Sobic.009G036500 | EE,MOE |
|  | iPGAM1 | Aco007146 | EE,CBS,G-box |  |  | PEQU_22778 | MOE | AT1G09780 | MOE,CBS | LOC_Os05g40420 | EE | Sobic.003G336000 | MOE |
|  | Nog1-2 | Aco018468 |  |  |  | PEQU_16017 | MOE | AT1G10300 |  | LOC_Os06g09570 |  | Sobic.010G073200 | MOE |
|  | PUB18 | Aco000945 | EE,G-box |  |  | PEQU_03171 | EE,MOE,CBS,G-box | AT1G10560 | G-box | LOC_Os08g32060 | G-box | Sobic.002G188700 | CBS,G-box |
|  | PWP2 | Aco006003 | MOE,CBS | CB5.v30038730 | MOE,CBS | PEQU_07670 | MOE | AT1G15440 |  | LOC_Os05g44320 |  | Sobic.009G201200 |  |
|  | ABA3 | Aco008369 | CBS | CB5.v30012280 |  | PEQU_31082 | MOE,G-box | AT1G16540 | G-box | LOC_Os06g45860 | MOE | Sobic.010G224000 |  |
|  | ROP2 | Aco008862 | EE,MOE | CB5.v30227970 |  | PEQU_03383 | MOE,G-box | AT1G20090 |  | LOC_Os02g20850 | CBS | Sobic.004G017900 | EE,MOE |
|  | BCA3 | Aco006181 | EE,MOE,CBS |  |  | PEQU_37822 | MOE | AT1G23730 | EE, |  |  | Sobic.003G234500 |  |
|  | RCN1 | Aco018967 | CBS | CB5.v30294160 |  | PEQU_22075 | MOE | AT1G25490 |  | LOC_Os09g07510 | MOE,CBS,G-box | Sobic.010G020400 |  |
|  | CIPK23 | Aco002215 | EE,G-box | CB5.v30252600 | MOE,CBS,G-box | PEQU_32901 | MOE | AT1G30270 | EE | LOC_Os07g05620 |  | Sobic.002G034700 | MOE |
|  | LrgB | Aco017000 | EE,MOE | CB5.v30122120 |  | PEQU_05821 | MOE,G-box | AT1G32080 |  | LOC_Os10g42780 | G-box | Sobic.001G283000 | EE |
|  | FBH3 | Aco000773 | MOE,CBS |  |  | PEQU_15028 | MOE,CBS | AT1G51140 | MOE,G-box | LOC_Os08g39630 | EE,MOE | Sobic.007G211000 | G-box |
|  | BGLU18 | Aco003169 | MOE |  |  | PEQU_19820 | MOE | AT1G52400 | G-box | LOC_Os08g39860 | MOE | Sobic.006G117400 | MOE,CBS,G-box |
|  | MYB50 |  |  |  |  |  |  | AT1G57560 | MOE | LOC_Os01g18240 |  |  |  |
|  | BCA6 | Aco002732 | CBS,G-box |  |  | PEQU_31387 | MOE,CBS | AT1G58180 | G-box | LOC_Os09g28910 | G-box | Sobic.002G230100 | EE,MOE |
|  | HT1 | Aco018941 | MOE | CB5.v30294410 | MOE | PEQU_12621 | MOE | AT1G62400 | G-box | LOC_Os06g43030 | EE | Sobic.010G200800 |  |
|  | RBOH_F | Aco005565 | CBS | CB5.v30048560 | MOE,CBS | PEQU_13986 | MOE | AT1G64060 | CBS | LOC_Os05g45210 |  | Sobic.003G287400 | G-box |
|  | FT | Aco010683 | MOE,CBS | CB5.v30100010 |  | PEQU_19304 | MOE | AT1G65480 |  | LOC_Os01g11940 |  | Sobic.003G017200 | G-box |
|  | ETR1 | Aco006353 | CBS | CB5.v30004070 | G-box | PEQU_04208 | MOE | AT1G66340 |  | LOC_Os03g49500 | MOE,CBS,G-box | Sobic.001G124200 | MOE,CBS |
|  | EXPA1 | Aco009615 | CBS | CB5.v30014600 | CBS | PEQU_28304 | MOE,CBS | AT1G69530 | MOE,CBS,G-box | LOC_Os05g39990 | MOE,G-box | Sobic.003G338801 |  |
|  | NPF4.6 | Aco026088 |  | CB5.v30068170 |  | PEQU_27578 | MOE,G-box | AT1G69850 |  | LOC_Os06g38294 |  | Sobic.010G175900 |  |
|  | BCA4 |  |  |  |  |  |  | AT1G70410 |  |  |  |  |  |
|  | EDS16 | Aco006849 |  |  |  | PEQU_20159 | MOE | AT1G74710 | MOE,CBS | LOC_Os09g19734 | MOE,CBS | Sobic.002G180400 | G-box |
|  | GLR2 | Aco013005 | MOE,CBS,G-box | CB5.v30144370 | MOE,G-box | PEQU_05546 | MOE | AT2G17260 | MOE | LOC_Os04g49570 | CBS | Sobic.010G231100 | EE,G-box |
|  | PLA2-BETA | Aco013004 | CBS | CB5.v30144360 | MOE | PEQU_07937 | MOE | AT2G19690 |  | LOC_Os02g58500 |  | Sobic.004G357800 | G-box |
|  | CCR2 | Aco004999 | EE,MOE,G-box |  |  | PEQU_11434 | MOE | AT2G21660 | EE | LOC_Os12g43600 | EE,MOE,G-box | Sobic.001G141300 | EE,MOE,G-box |
|  | ELF3 | Aco005852 | CBS |  |  | PEQU_13253 | MOE | AT2G25930 |  | LOC_Os06g05060 |  | Sobic.003G191700 | EE |
|  | GP_ALPHA_1 | Aco003966 |  | CB5.v30084430 |  | PEQU_25983 | MOE | AT2G26300 | MOE | LOC_Os05g26890 | EE,MOE,G-box | Sobic.001G484200 | EE,G-box |
|  | TED4 | Aco014271 |  | CB5.v30059900 |  | PEQU_15541 | MOE | AT2G26670 | CBS | LOC_Os06g40080 | CBS,G-box | Sobic.010G184600 | EE,MOE |
|  | SCAB1 | Aco007230 | CBS | CB5.v30010250 | CBS | PEQU_18950 | MOE | AT2G26770 |  | LOC_Os01g59130 |  | Sobic.003G328600 | MOE |
|  | DOR | Aco000868 | MOE,G-box |  |  | PEQU_00038 | EE,MOE,CBS | AT2G31470 | EE | LOC_Os09g20650 | MOE,CBS, | Sobic.008G056200 | EE,MOE |
|  | COP1 | Aco001355 | MOE |  |  | PEQU_18985 | EE,MOE,CBS | AT2G32950 | MOE | LOC_Os02g53140 | MOE, | Sobic.004G312600 | EE,G-box |
|  | RD20 | Aco019722 | G-box | CB5.v30255070 |  | PEQU_33467 | MOE | AT2G33380 | G-box | LOC_Os04g43200 | MOE,G-box | Sobic.006G143600 |  |
|  | COI1 | Aco014112 |  | CB5.v30092400 | CBS | PEQU_09359 | MOE | AT2G39940 |  | LOC_Os05g37690 | EE | Sobic.009G157200 | EE,MOE |
|  | ABI4 | Aco000059 |  | CB5.v30054360 | G-box | PEQU_10543 | MOE | AT2G40220 |  | LOC_Os05g28350 | MOE | Sobic.009G103800 |  |
|  | JAR1 | Aco005530 | EE,MOE | CB5.v30185440 | MOE | PEQU_33996 | EE,MOE,G-box | AT2G46370 | MOE,CBS,G-box | LOC_Os05g50890 | EE,G-box | Sobic.009G249900 | MOE |
|  | ABCC4 | Aco017858 | EE,CBS | CB5.v30212050 | CBS | PEQU_01441 | MOE,G-box | AT2G47800 | G-box | LOC_Os01g25386 | MOE,G-box | Sobic.003G160200 | MOE,G-box |
|  | CA1 |  |  |  |  |  |  | AT3G01500 |  | LOC_Os01g45274 | EE,MOE,CBS, |  |  |
|  | iPGAM2 |  |  | CB5.v30119590 | MOE,G-box |  |  | AT3G08590 |  |  |  |  |  |
|  | LOG2 | Aco017758 | MOE | CB5.v30124790 | MOE | PEQU_14893 | EE,MOE | AT3G09770 | MOE,CBS | LOC_Os03g15000 | EE | Sobic.001G433400 | G-box |
|  | CHC1 | Aco000555 |  | CB5.v30035580 |  | PEQU_13765 | MOE | AT3G11130 |  | LOC_Os12g01390 |  | Sobic.005G003300 | MOE |
|  | PP2CA | Aco016843 | G-box |  |  | PEQU_41297 | MOE,G-box | AT3G11410 | EE,CBS,G-box |  |  | Sobic.003G354000 | MOE,G-box |
|  | SYP121 | Aco005123 | CBS | CB5.v30059110 | MOE,G-box | PEQU_39338 | MOE,CBS | AT3G11820 | EE,MOE | LOC_Os06g39050 |  | Sobic.010G177500 | G-box |
|  | PLDALPHA1 | Aco014266 | EE,G-box | CB5.v30244270 | MOE | PEQU_08286 | MOE,G-box | AT3G15730 | MOE | LOC_Os01g07760 | G-box | Sobic.003G050400 | G-box |
|  | PHOT1 | Aco000718 |  | CB5.v30035240 | MOE,CBS,G-box | PEQU_36161 | MOE | AT3G45780 | MOE | LOC_Os12g01140 |  | Sobic.008G001000 |  |
|  | PME34 | Aco010826 | MOE | CB5.v30164990 | MOE,G-box | PEQU_09085 | MOE | AT3G49220 | MOE | LOC_Os01g13320 |  | Sobic.003G148400 | MOE |
|  | CEN2 | Aco004005 | EE | CB5.v30084840 | G-box | PEQU_34003 | MOE | AT3G50360 | MOE,G-box | LOC_Os10g25010 |  | Sobic.002G376500 |  |
|  | FER | Aco018669 |  | CB5.v30192930 |  | PEQU_02509 | EE,MOE | AT3G51550 | EE | LOC_Os01g56330 | G-box | Sobic.001G382400 |  |
|  | PIP5K4 | Aco021109 | EE | CB5.v30192740 |  | PEQU_08680 | MOE | AT3G56960 | EE | LOC_Os07g46490 | EE | Sobic.002G403100 | EE,G-box |
|  | MYB55 |  |  |  |  |  |  | AT4G01680 |  |  |  |  |  |
|  | TPC1 |  |  | CB5.v30233370 | MOE | PEQU_26772 | MOE,CBS | AT4G03560 | G-box | LOC_Os01g48680 | G-box | Sobic.003G256100 | EE |
|  | CRY1 | Aco001216 | MOE,CBS | CB5.v30164550 | CBS,G-box | PEQU_07192 | MOE,CBS,G-box | AT4G08920 | EE,MOE,CBS | LOC_Os04g37920 | G-box | Sobic.006G101600 | MOE |
|  | RR2 | Aco013421 |  | CB5.v30137390 | MOE,G-box | PEQU_16180 | MOE,CBS | AT4G16110 | CBS | LOC_Os03g12350 | MOE,G-box | Sobic.001G451000 |  |
|  | CBL1 | Aco011645 | G-box |  |  | PEQU_01331 | MOE,G-box | AT4G17615 | EE,MOE,G-box | LOC_Os10g41510 | CBS,G-box | Sobic.001G294300 | EE,G-box |
|  | ALMT12 | Aco012924 | CBS | CB5.v30023110 | CBS | PEQU_22847 | MOE | AT4G17970 | CBS,G-box | LOC_Os10g42180 | EE,G-box | Sobic.010G220700 | MOE |
|  | KAT2 | Aco010657 | MOE,G-box | CB5.v30204160 |  | PEQU_34686 | MOE | AT4G18290 | EE | LOC_Os02g14840 | MOE,CBS,G-box | Sobic.004G107500 |  |
|  | BHP | Aco006025 | G-box | CB5.v30048860 | G-box | PEQU_33458 | MOE | AT4G18950 | MOE,G-box | LOC_Os01g54480 |  | Sobic.003G295200 |  |
|  | TSF |  |  |  |  | PEQU_06787 | MOE,G-box | AT4G20370 | MOE, |  |  | Sobic.010G045100 | MOE,CBS,G-box |
|  | GHR1 | Aco005069 |  | CB5.v30171870 |  | PEQU_02924 | EE,MOE,G-box | AT4G20940 |  | LOC_Os07g05190 |  | Sobic.002G032500 | MOE |
|  | CDPK6 | Aco006066 |  | CB5.v30038050 |  | PEQU_10718 | MOE | AT4G23650 | G-box | LOC_Os01g43410 | EE,MOE | Sobic.009G249000 |  |
|  | NLP7 | Aco004459 | CBS,G-box | CB5.v30179830 | CBS,G-box | PEQU_02529 | MOE | AT4G24020 | CBS | LOC_Os01g13540 | MOE,G-box | Sobic.003G003600 | MOE,G-box |
|  | ABI1 | Aco005893 | G-box |  |  | PEQU_00876 | MOE | AT4G26080 |  | LOC_Os01g40094 | CBS,G-box | Sobic.003G198200 | MOE |
|  | VAMP711 | Aco011520 | MOE,CBS,G-box | CB5.v30133080 |  | PEQU_24339 | MOE | AT4G32150 | MOE,G-box | LOC_Os06g07780 |  | Sobic.010G058500 |  |
|  | EDA39 | Aco009926 | MOE,G-box | CB5.v30175560 | MOE,G-box | PEQU_13247 | MOE | AT4G33050 | MOE | LOC_Os01g38980 |  | Sobic.003G193800 | MOE |
|  | BCA5 |  |  | CB5.v30091940 | CBS,G-box |  |  | AT4G33580 | MOE | LOC_Os09g28910 | G-box |  |  |
|  | AGB1 | Aco008643 | G-box | CB5.v30118900 | G-box | PEQU_33700 | MOE,G-box | AT4G34460 |  | LOC_Os03g46650 | MOE,G-box | Sobic.001G142100 | EE,CBS |
|  | PRSL1 | Aco001264 | MOE,CBS,G-box | CB5.v30247170 | CBS,G-box | PEQU_24705 | MOE,CBS | AT4G40100 |  | LOC_Os06g11430 | G-box | Sobic.001G477300 |  |
|  | EIN2 | Aco005120 | MOE,G-box |  |  | PEQU_40634 | MOE,G-box | AT5G03280 | G-box | LOC_Os07g06130 | MOE | Sobic.003G014500 | MOE,CBS,G-box |
|  | UGT76C2 | Aco000490 | MOE,G-box |  |  | PEQU_11226 | EE,MOE | AT5G05860 | G-box | LOC_Os07g13800 | G-box | Sobic.002G085400 |  |
|  | PATROL1 | Aco004762 | G-box | CB5.v30165760 |  | PEQU_17240 | MOE | AT5G06970 | G-box | LOC_Os03g47930 | MOE | Sobic.009G118900 |  |
|  | OCP3 | Aco014285 |  | CB5.v30059760 |  | PEQU_23420 | MOE | AT5G11270 |  | LOC_Os06g39906 | EE | Sobic.010G183400 |  |
|  | BCA2 |  |  | CB5.v30069370 |  |  |  | AT5G14740 |  |  |  |  |  |
|  | MPC1 | Aco000930 | EE | CB5.v30008550 |  | PEQU_05217 | MOE | AT5G20090 |  | LOC_Os09g20660 | G-box | Sobic.002G186500 | G-box |
|  | PHS1 | Aco002492 | MOE | CB5.v30152560 |  | PEQU_23661 | MOE | AT5G23720 | MOE | LOC_Os01g20940 | EE,G-box | Sobic.003G147500 | G-box |
|  | TGG2 |  |  |  |  |  |  | AT5G25980 | EE | LOC_Os04g39864 | EE,G-box |  |  |
|  | TGG1 |  |  |  |  |  |  | AT5G26000 | EE |  |  |  |  |
|  | MYB86 | Aco022092 | MOE | CB5.v30287100 |  | PEQU_05119 | MOE | AT5G26660 |  |  |  |  |  |
|  | RAN1 | Aco006600 | MOE,CBS | CB5.v30037280 | MOE,CBS | PEQU_29215 | MOE | AT5G44790 | CBS | LOC_Os02g07630 | G-box | Sobic.004G057400 |  |
|  | VAM3 | Aco016027 | EE | CB5.v30186060 |  | PEQU_20870 | MOE,CBS | AT5G46860 | CBS,G-box | LOC_Os01g15110 | MOE,G-box | Sobic.003G116600 |  |
|  | CBL9 |  |  | CB5.v30196080 | G-box |  |  | AT5G47100 | EE,MOE,CBS,G-box |  |  |  |  |
|  | LHCB3 | Aco009134 | CBS | CB5.v30189230 | CBS | PEQU_29347 | MOE | AT5G54270 |  | LOC_Os07g37550 | CBS | Sobic.002G339200 | G-box |
|  | KCO1 | Aco017556 | EE | CB5.v30031660 |  | PEQU_27968 | MOE | AT5G55630 | CBS | LOC_Os03g54100 |  | Sobic.001G086900 | G-box |
|  | PHOT2 |  |  | CB5.v30247620 |  | PEQU_15152 | EE,MOE | AT5G58140 | MOE |  |  | Sobic.007G105500 |  |
|  | HAI1 |  |  | CB5.v30123820 | CBS,G-box |  |  | AT5G59220 | EE,CBS,G-box |  |  | Sobic.001G424400 | CBS |
|  | SCAP1 | Aco030448 |  |  |  | PEQU_13748 | MOE,CBS | AT5G65590 |  | LOC_Os07g13260 | G-box | Sobic.001G034300 |  |

**Supplementary Table S2.** Enriched *cis*-regulating motifs in CAM species and non-CAM species

| **Photosynthetic type** | **Motif ID in *Arabidopsis* DAP database** | **Motif Name** | **Consensus sequences^c^** |
| --- | --- | --- | --- |
| CAM^a^ | AP2EREBP_tnt.ERF73_col_a_m1 | ERF73 | YCCCGCCGCCRYHDYHN |
|  | AP2EREBP_tnt.ERF7_colamp_a_m1 | ERF7 | ATGGCGGCGGHRRNRRNDRND |
|  | AP2EREBP_tnt.ABR1_col_a_m1 | ABR1 | GRHGRHDDTKGCGGCGGMG |
| C_3_ or C_4_^b^ | HMG_tnt.3XHMGBOX1_col_a_m1 | 3XHMGBOX1 | RCGGCGGHGRWDRNGGNRRNG/ WCCACCACCACCWCC |
|  | LOBAS2_tnt.LOB_col_a_m1 | LOB | WCCGCCGCCDYCKCCGCCGCH |

^a^ Photosynthetic type CAM includes *A. comosus* var. *comosus*, *A. comosus* var. *bracteatus* and *P. equestris*

^b^ Photosynthetic type C_3_ or C_4_ include *Arabidopsis*, rice, and sorghum

^c^ Consensus sequences were constructed by CentriMo program by using the "50% rule"

**Supplementary Table S3. List of *A. comosus* var. *comosus* gene names and gene IDs used in this perspective.**

| **Gene** | **Gene name** | **Gene ID** |
| --- | --- | --- |
| *CA* | *AccαCA1-1* | Aco007803 |
|  | *AccαCA1-2* | Aco016727 |
|  | *AccαCA7* | Aco001338 |
|  | *AccβCA5* | Aco002732 |
|  | *AccβCA2-1* | Aco005402 |
|  | *AccβCA2-2* | Aco006181 |
|  | *AccγCA1-1* | Aco014975 |
|  | *AccγCA1-2* | Aco023760 |
|  | *AccγCA2L* | Aco019038 |
| *PPC* | *AccPPCH1* | Aco010025 |
|  | *AccPPCH2* | Aco018093 |
|  | *AccPPC4* | Aco016429 |
| *PPCK* | *AccPPCK1* | Aco010095 |
|  | *AccPPCK2* | Aco013938 |
| *MDH* | *AccMDH1* | Aco002885 |
|  | *AccMDH2* | Aco004349 |
|  | *AccMDH3* | Aco004996 |
|  | *AccMDH4* | Aco006122 |
|  | *AccMDH5* | Aco007734 |
|  | *AccMDH6* | Aco008626 |
|  | *AccMDH7* | Aco010232 |
|  | *AccMDH8* | Aco013935 |
|  | *AccMDH9* | Aco014690 |
|  | *AccMDH10* | Aco017525 |
|  | *AccMDH11* | Aco017526 |
|  | *AccMDH12* | Aco017527 |
|  | *AccMDH13* | Aco017528 |
|  | *AccMDH14* | Aco019631 |
| *ALMT* | *AccALMT1-1* | Aco007539 |
|  | *AccALMT1-2* | Aco015592 |
|  | *AccALMT1-3* | Aco016774 |
|  | *AccALMT1-4* | Aco022068 |
|  | *AccALMT1-5* | Aco028383 |
|  | *AccALMT9-1* | Aco003023 |
|  | *AccALMT9-2* | Aco005552 |
|  | *AccALMT9-3* | Aco010725 |
| *DT* | *AccDT1* | Aco000795 |
|  | *AccDT2* | Aco006063 |
|  | *AccDT3* | Aco006064 |
|  | *AccDT4* | Aco010756 |
|  | *AccDT5* | Aco015541 |
| *NAD_ME* | *AccNAD-ME1* | Aco007622 |
|  | *AccNAD-ME2* | Aco016569 |
| *NADP_ME* | *AccNADP-ME1* | Aco005631 |
|  | *AccNADP-ME2* | Aco005989 |
|  | *AccNADP-ME3* | Aco009967 |
| *PCK* | *AccPCK1* | Aco017762 |
| *PPDK* | *AccPPDK1* | Aco024818 |
